# Differences in physical fitness levels by adherence to the 24-hour movement guidelines among Japanese elementary school children

**DOI:** 10.1371/journal.pone.0337972

**Published:** 2025-12-03

**Authors:** Takashi Naito, Kenryu Aoyagi, Koichiro Oka, Kaori Ishii

**Affiliations:** 1 Graduate School of Sport Sciences, Waseda University, Tokorozawa, Saitama, Japan; 2 Organization for the Strategic Coordination of Research and Intellectual Properties, Meiji University, Chiyoda-Ku, Tokyo, Japan; 3 College of Economics, Kanto Gakuin University, Kanazawa-ku, Kanagawa, Japan; 4 Faculty of Sport Sciences, Waseda University, Tokorozawa, Saitama, Japan; Saga University, JAPAN

## Abstract

Currently, physical fitness levels of Japanese children are lower than in the 1980s. Investigating the relationship between adherence to the 24-hour movement guidelines (24-h MG)—which include moderate-to-vigorous physical activity (MVPA), screen time (ScT), and sleep duration (Sleep)—and physical fitness is crucial for improving children’s fitness. This cross-sectional study examined differences in physical fitness by 24-h MG adherence patterns among children in grades 1–6. Eight fitness components were assessed using standardized tests: handgrip strength (muscle strength), sit-up (trunk muscle strength and endurance), sit-and-reach (flexibility), repeated side jump (agility), 20-meter shuttle run (cardiorespiratory fitness), 50-meter sprint (speed), standing long jump (explosive power), and softball throw (explosive power and dexterity). A total of 307 participants (41.4% male) were included in the analysis. Analysis of covariance was performed, with sex, grade, BMI, and other guideline adherence as covariates. Total fitness scores were significantly higher in those meeting the MVPA (Cohen’s d = 0.47; standardized effect size), both the MVPA and ScT (d = 0.63), both the MVPA and Sleep guidelines (d = 0.65), or with all three guidelines (d = 0.59) compared to those not meeting them. Children meeting the MVPA guideline—either alone or in combination with ScT or Sleep guidelines—showed significantly higher scores in multiple fitness components compared to those not meeting them. When comparing effect sizes, differences in total and most of the individual fitness scores were greater among those who met both the MVPA and either the ScT or Sleep guideline. In contrast, those who did not meet the MVPA guideline but adhered to one or both of the ScT and Sleep guidelines showed no significant differences in total and individual fitness scores. These findings suggest that promoting MVPA adherence is crucial for enhancing physical fitness, and that additionally encouraging appropriate ScT and Sleep behaviors may further improve children’s physical fitness.

## Introduction

Previous research has revealed that 81% of children and adolescents worldwide are physically inactive [1] and spend an excessive portion of their time in sedentary behavior [2,3]. These lifestyles may have partially contributed to the observed decline in physical fitness among children over the past few decades [4–8]. Similarly, trends have been reported in Japan, where the physical fitness of students in elementary schools declined compared to the 1980s [9,10]. Contributing factors include a reduction in time spent engaging in physical activity and outdoor play in daily life [11]. Revisions to the Guidelines for the Course of Study also reduced the number of physical education classes in elementary schools and shifted the focus from physical fitness to enjoyment [12]. The development of physical fitness in childhood is critical, as it is associated with both physical and mental health outcomes during childhood [13,14], as well as mental health and cognitive performance during adolescence [15].

Previous studies have revealed associations between children’s physical fitness levels and a range of lifestyle behaviors, including physical activity, sedentary behavior, and sleep [13,16–19]. The concept of 24-hour movement guidelines (24-h MG), which aim to promote these three movement behaviors in an integrated manner rather than individually, has become increasingly widespread internationally [20–24]. The 24-h MG framework encompasses physical activity, sedentary behavior, and sleep within a 24-h period, during which these behaviors complement each other. In Canada and Australia, 24-h MG are recommended for children aged 5–17 years, as follows: (1) 60 min of daily moderate-to-vigorous physical activity (MVPA), (2) no more than 2 hours of screen time (ScT) per day (excluding schoolwork), and (3) 9–11 h of sleep duration (Sleep) for children aged 5–13 years, and 8–10 h for those aged 14–17 years [20,25].

Several previous studies have revealed associations between adherence to the 24-h MG and physical fitness, with greater adherence linked to improved aerobic capacity, muscular strength, and total physical fitness [26–28]. A systematic review revealed that adherence to the MVPA component of the 24-h MG was associated with higher physical fitness levels [29]. Associations between various patterns of MVPA, ScT, and Sleep adherence and physical fitness have also been investigated [26–28]. A previous study among Japanese elementary school children examined four physical fitness components—grip strength, sit-ups, sit-and-reach, and the 20-meter shuttle run—and found that adherence to specific 24-h MG combinations was linked to higher muscular and cardiorespiratory fitness [30]. However, research involving children remains limited, particularly in exploring the relationship between adherence to the 24-h MG and a wider range of fitness components—such as agility, speed, explosive power, and dexterity. A systematic review indicates that higher levels of fundamental movement skills (e.g., running, jumping, throwing, balancing), beyond specific physical fitness components, are positively associated with physical activity outcomes, including participation in organized sports and daily step counts, among children and adolescents [31]. These findings highlight the need to assess diverse physical fitness elements and to identify associated modifiable lifestyle factors (e.g., physical activity, sedentary behavior, and sleep duration) in order to better understand and support children’s comprehensive physical development.

In Japan, although a general decline in children’s physical fitness compared to the 1980s has been reported [10], this trend has not occurred uniformly across all physical fitness measurement categories. For comparable physical fitness test items and age groups, compared to those in 1980, performance in the 50-meter sprint among 7-, 9-, and 11-year-old children declined by approximately 3% for both boys and girls by 2021. Moreover, a substantial decline of 20–25% was observed in the softball throw. At age 11, handgrip strength decreased by 5.7% in boys and 3.5% in girls compared to 1980, whereas the repeated side jump at the same age improved by approximately 11% [32,33]. Thus, investigating the association between 24-h MG and individual as well as total physical fitness components is critical when considering strategies to improve children’s physical fitness. The 24-h MG itself provides a comprehensive behavioral framework that integrates physical activity, screen time, and sleep as interrelated components of daily behavior. Applying this framework may provide preliminary insights into why certain fitness components have declined, whereas others have improved or remained stable. Understanding the specific relationships between adherence to each guideline—MVPA, ScT, and Sleep—and individual fitness components may support the development of more targeted and effective strategies for reversing these declines and promoting balanced physical development among children.

The purpose of this study was to comprehensively investigate the relationship between 24-h MG and both the total physical fitness scores and individual component scores, using a standardized eight-item physical fitness test [34], which is implemented in elementary schools across Japan.

## Materials and methods

### Participants

This study was conducted on a cohort of children enrolled in grades 1–6 at an elementary school in Yokohama City, Japan. In 2021, a total of 477 children (222 boys and 255 girls), aged 6–11 years, were included in the study. Participants were recruited from a cooperating school between April 1 and 30, 2021. Written informed consent to participate in the study was obtained from both the children and their parents. The study was approved by the Waseda University Ethics Review Procedures Concerning Research with Human Subjects (approval numbers: 2018−191 and 2024-HN032). All research procedures adhered to the Declaration of Helsinki and the Ethical Guidelines for Medical and Biological Research Involving Human Subjects established by the Japanese government. The questionnaire was administered in a paper-based format. For the self-reported activity behavior questionnaire, children in grades 4 and above are generally able to provide reliable responses [35]. Therefore, children in grades 1–3 completed the questionnaire with assistance from their parents, while children in grades 4–6 completed the survey by themselves. The questionnaire was designed to be completed in approximately 5–10 minutes.

### MVPA survey

MVPA was assessed using an originally developed questionnaire based on a previous study [36] and has also been used in prior research involving Japanese elementary school students of the same age [37]. Although it is widely agreed that children should engage in at least 60 min of moderate-to-vigorous physical activity (MVPA) daily, no precise assessment scale has been established in Japan. Therefore, participants were asked whether they engaged in MVPA that induces heavier-than-usual breathing—such as walking, cycling, brisk walking, dancing, or swimming, football, or basketball—for at least 60 min per day, including physical education, recess, and other daily activities. If so, they were then asked how many days they engaged in MVPA during a typical week. Children who reported physical activity for at least 60 min per day on 7 days per week were classified as meeting the MVPA guideline [38,39], whereas those who performed such activity for 6 days or fewer were classified as not meeting the MVPA guideline.

### Survey of screen time

Recreational screen time was assessed using a standard questionnaire [40] covering these three types of screen use: (1) watching television or videos, (2) playing video games (including all console-based video games, mobile games, or computer games), and (3) using the internet or e-mail for purposes other than schoolwork outside of class time. Participants were asked to report the number of days, and the average duration per day spent on each activity during weekdays and weekends, based on their usual behavior over a typical week. The total weekday and weekend screen times were summed and divided by seven to calculate the average daily screen time. Children with an average screen time of ≤ 2 h per day were classified as meeting the ScT guideline [20,25], and those exceeding 2 h per day were classified as not meeting the ScT guideline.

### Survey of sleep duration

Sleep duration was assessed using an originally developed questionnaire based on the sleep duration criteria of the 24-h MG [20,25]. Participants were asked to select one of three response options regarding their usual night-time sleep duration: (1) < 9 h, (2) 9–11 h, or (3) ≥ 11 h. Children reporting 9–11 h of sleep were classified as meeting the Sleep guideline, whereas those reporting < 9 h and ≥ 11 h were classified as not meeting the Sleep guideline.

### Measurement of physical fitness

Physical fitness test scores were obtained from the elementary school. The physical fitness assessment comprised eight components: handgrip strength as a measure of muscle strength, sit-up as an indicator of trunk muscle strength and endurance, sit-and-reach as a measure of flexibility, repetitive side jump as an assessment of agility, a 20-meter shuttle run as an evaluation of cardiorespiratory fitness, a 50-meter sprint as a test of speed, a standing long jump as a measure of explosive power, and a softball throw as an assessment of explosive power and dexterity [41]. All physical fitness tests were conducted in accordance with the guidelines of the New Physical Fitness Test Implementation Guidelines for children aged 6–11 years issued by the Ministry of Education, Culture, Sports, Science and Technology (MEXT), Japan [34]. Since this guideline does not specify who should conduct the measurements, the procedures at the participating school were applied flexibly depending on factors such as grade level, test item, and the condition of the children. Therefore, it was difficult to specify whether these measures were assessed by the teacher or by the children evaluating each other (e.g., by having a partner child count the number of side steps in the repeated side jump test).

The Handgrip strength was measured using a Smedley-type dynamometer. Participants performed two trials with each hand while standing, alternating between right and left hands, and the better score from each hand was averaged. The Sit-up was performed with the participant lying on a mat, knees bent at 90 degrees, and arms crossed in front of the chest. A partner held the participant’s feet to keep them in place. Participants repeatedly raised their upper body so that their elbows touched their thighs, then returned to the starting position. The number of correctly performed sit-ups in 30 seconds was recorded. The Sit-and-reach was performed in a seated position with the participant’s back and hips touching a wall and both legs extended forward. Participants placed both hands on top of a standardized cardboard box and bent forward without bending their knees, sliding the entire box forward. The distance the box moved (in cm) was measured. Two trials were conducted, and the better score was used. The Repetitive side jump was performed between two parallel lines placed 100 cm to the left and right of a central line. Starting from the central line, participants repeatedly performed side-steps to touch or cross each line alternately without jumping, for 20 seconds. Two trials were conducted, and the higher score was used. The 20-meter shuttle run was performed with participants starting to run at the sound of a beep played from a CD. They ran to a line 20 meters away and turned around after touching or crossing it with one foot before the next beep. This was repeated back and forth. The interval between beeps gradually shortened, approximately every minute, according to the standardized protocol, requiring increased running speed. The test ended when the participant failed to reach the line in time for two consecutive beeps or could no longer continue. The total number of completed 20-meter runs was recorded. The 50-meter sprint was performed from a standing start along a straight course. The time was recorded to the nearest 0.1 seconds, with any time under 0.1-second increments rounded up. The standing long jump was performed with participants standing so that their toes were aligned with the front edge of the take-off line, and then jumping forward as far as possible. The longer distance from two trials was recorded. The softball throw was performed with participants throwing a regulation-size softball from within a marked circle. The distance to the point of initial ground contact was measured, and any value below one meter was rounded down to the nearest whole meter. The better of two trials was recorded.

Each component was scored on a 10-point scale (1–10) using standardized criteria of New Physical Fitness Test Implementation Guidelines (S1 Table) [34]. A total physical fitness score was calculated by summing the eight component scores, yielding a possible range of 8–80 points.

### Statistical analysis

Of the 477 participants initially enrolled, 307 children (64.4%) were included in the final analysis. The remaining 170 participants (approximately 36%) were excluded due to missing data on key variables, including physical fitness measures or responses to the MVPA, ScT, or Sleep questionnaires. The missing data were primarily due to absences on testing days, incomplete measurements, or non-responses to questionnaires. No data imputation was performed; only complete cases were included in the analysis. Participants’ characteristics and adherence status to the 24-h MG were analyzed using descriptive statistics. Body mass index (BMI) and physical fitness scores were described as mean ± standard deviation. Differences in physical fitness scores by 24-h MG adherence were examined using analysis of covariance (ANCOVA). The primary independent variable was adherence to the target 24-h MG pattern (e.g., MVPA, MVPA and ScT, etc.). Covariates also included sex, grade, BMI, and adherence to the remaining 24-h MG components not specified as the target were included as covariates.

Comparisons were conducted between children who met a specific recommendation pattern and those who did not. For example, when comparing children who met both the MVPA and ScT guidelines with those who did not, the “meet” group (n = 21) included children who adhered to both the MVPA and ScT or to all three guidelines. The “do not meet” group (n = 286) included all other adherence patterns, such as meeting MVPA only, ScT only, Sleep only, MVPA and Sleep, ScT and Sleep, or none of the three. Residual normality was visually assessed using Q–Q plots, and no substantial deviations were observed. Homoscedasticity was evaluated using Levene’s test. For outcomes in which the assumption of homoscedasticity was violated, P-values were calculated using heteroskedasticity-consistent (HC3) standard errors. Standardized effect sizes were calculated as approximate Cohen’s d by dividing the adjusted mean difference from the ANCOVA by the residual standard deviation [42,43]. Effect sizes were interpreted as 0.2, 0.5, and 0.8 representing small, medium, and large effects, respectively [44]. A significance level was set at P < 0.05. All statistical analyses were performed using SPSS (version 29.0; IBM Corp., Armonk, NY, USA).

## Results

**Fig 1:**
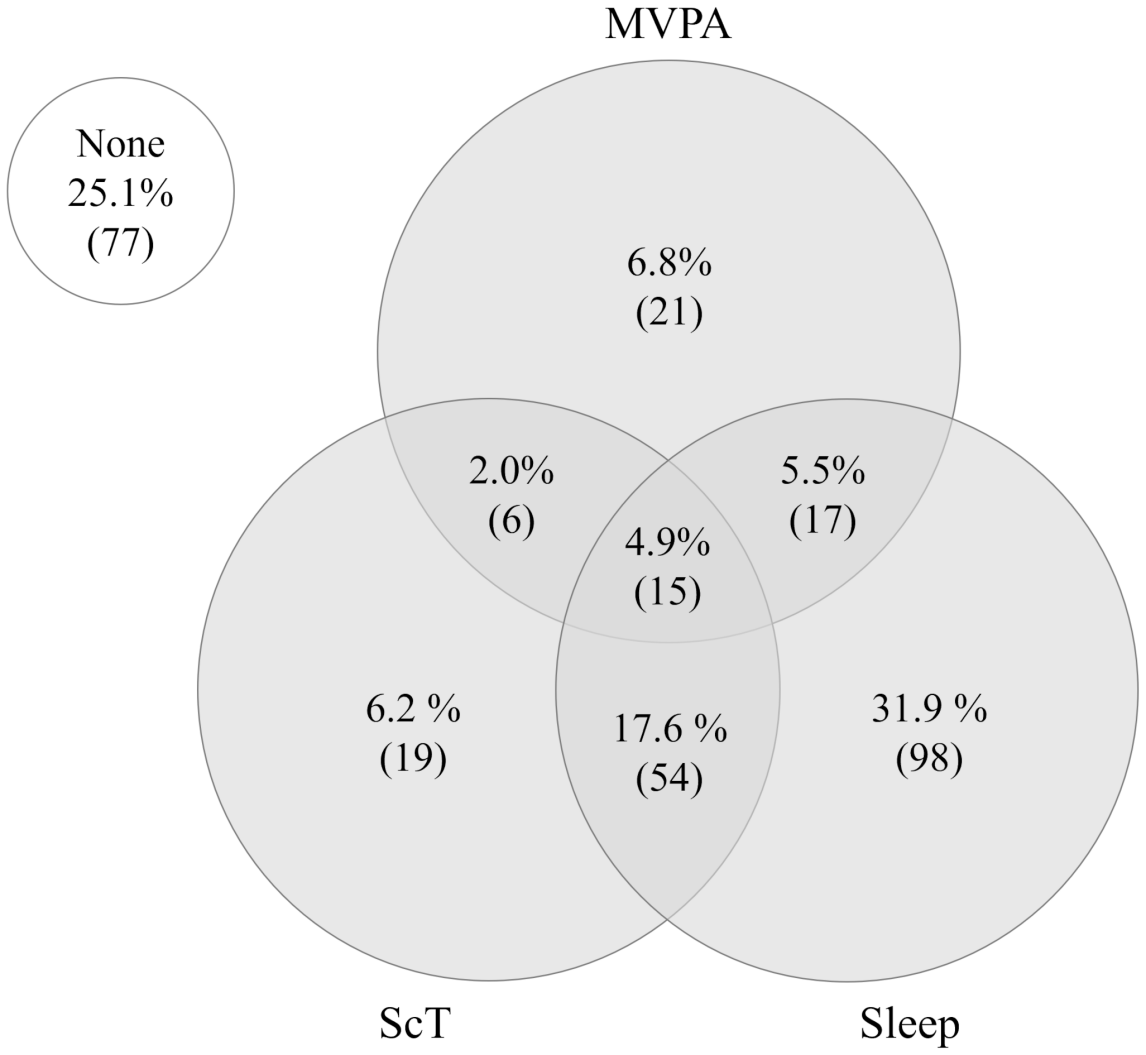
Status of adherence to the 24-hour movement guidelines. Venn diagram illustrating the proportion and the corresponding number of children (shown in parentheses) in the full sample (n = 307) meeting none of the guidelines, each individual guideline for moderate-to-vigorous physical activity (MVPA), screen time (ScT), and sleep duration (Sleep), as well as combinations of these guidelines.

Table 1 summarizes the characteristics of the participants. A total of 307 participants (41.4% male) were included in the final analysis after excluding those with missing data. Table 2 presents the results of the physical fitness tests; the mean (±SD) total fitness score was 44.5 ± 12.0. Fig 1 shows adherence rates for MVPA, ScT, and Sleep according to the 24-h MG. In this study, 4.9% (n = 15) of participants met all three recommendations, whereas 25.1% (n = 77) did not meet any.

**Table 1:** Characteristics of participants.

Variable		N	%
Sex	Male	127	41.4
	Female	180	58.6
Grade	1st Grade	38	12.4
	2nd Grade	40	13.0
	3rd Grade	38	12.4
	4th Grade	49	16.0
	5th Grade	65	21.2
	6th Grade	77	25.1
Body mass index	mean ± SD	17.1	2.7

**Table 2:** Total fitness score and eight physical fitness component scores.

Variable	Mean	SD
Total fitness score	44.5	12.0
Handgrip strength	5.4	1.7
Sit-up	6.5	1.8
Sit-and-reach	6.0	2.2
Repetitive side jump	5.0	2.1
20-meter shuttle run	5.5	2.0
50-meter sprint	5.8	1.9
Standing long jump	5.4	2.0
Softball throw	5.0	1.8

The total physical fitness score ranged from 8 to 80, and each physical fitness component score ranged from 1 to 10.

**Fig 2:**
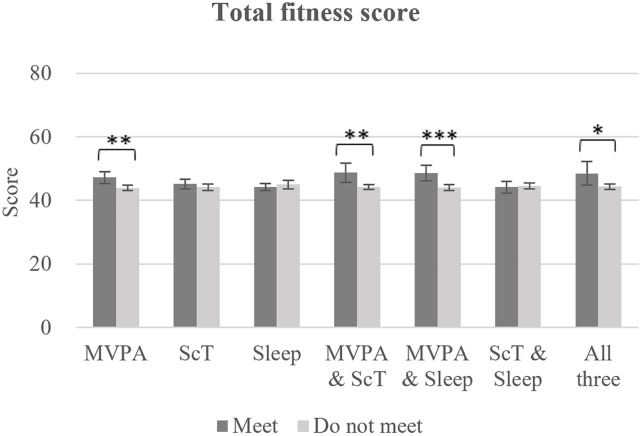
Differences in total physical fitness scores by 24-hour movement guideline adherence patterns. Comparisons were conducted between children who met a specific recommendation pattern and those who did not. For the labels “MVPA,” “ScT,” and “Sleep,” each category includes all children meeting the respective guideline, regardless of whether they also met the other guidelines; adherence to the other guidelines was included as covariates in the model to minimize potential confounding. Error bars show 95% confidence intervals. Asterisks indicate statistical significance: P < 0.05 (*), < 0.01 (**), < 0.001 (***). Sample sizes for each group were as follows: MVPA (meet: n = 59, do not meet: n = 248), ScT (meet: n = 94, do not meet: n = 213), Sleep (meet: n = 184, do not meet: n = 123), MVPA and ScT (meet: n = 21, do not meet: n = 286), MVPA and Sleep (meet: n = 32, do not meet: n = 275), ScT and Sleep (meet: n = 69, do not meet: n = 238), All three (meet: n = 15, do not meet: n = 292). As an example, for the MVPA and ScT combination, the “do not meet” group (n = 286) included all other adherence patterns, such as meeting MVPA only, ScT only, Sleep only, MVPA and Sleep, ScT and Sleep, or none of the three.

**Fig 3:**
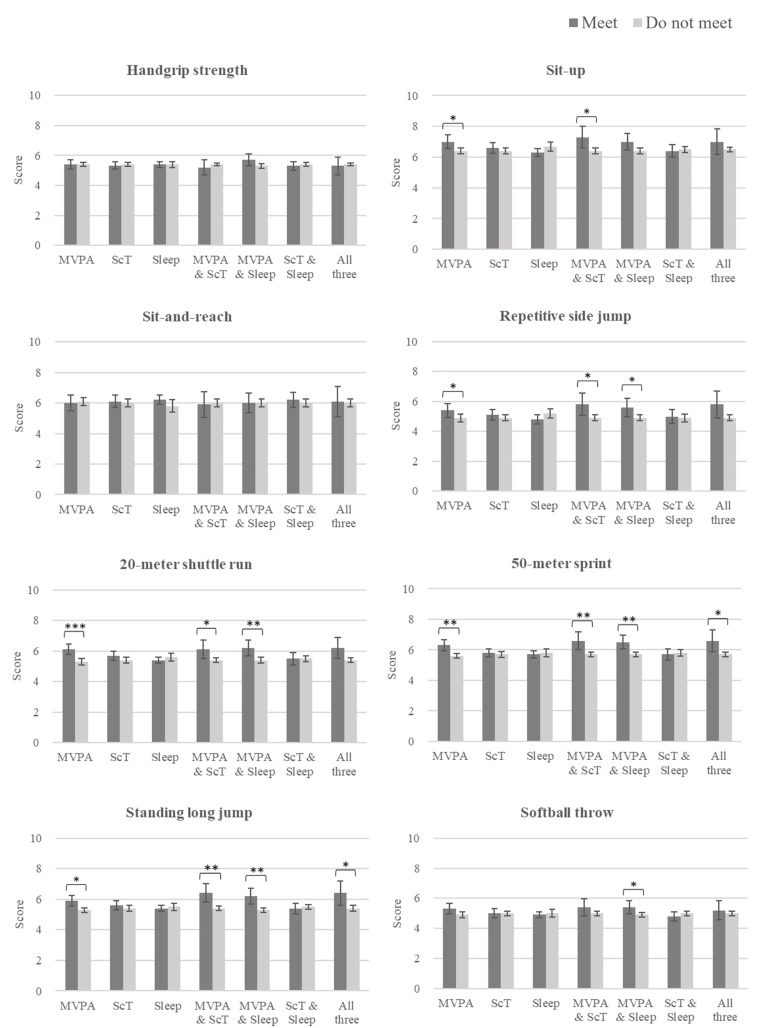
Differences in individual physical fitness component scores by 24-hour movement guideline adherence patterns. Comparisons were conducted between children who met a specific recommendation pattern and those who did not. For the labels “MVPA,” “ScT,” and “Sleep,” each category includes all children meeting the respective guideline, regardless of whether they also met the other guidelines; adherence to the other guidelines was included as covariates in the model to minimize potential confounding. Error bars show 95% confidence intervals. Asterisks indicate statistical significance: P < 0.05 (*), < 0.01 (**), < 0.001 (***). Sample sizes for each group were as follows: MVPA (meet: n = 59, do not meet: n = 248), ScT (meet: n = 94, do not meet: n = 213), Sleep (meet: n = 184, do not meet: n = 123), MVPA and ScT (meet: n = 21, do not meet: n = 286), MVPA and Sleep (meet: n = 32, do not meet: n = 275), ScT and Sleep (meet: n = 69, do not meet: n = 238), All three (meet: n = 15, do not meet: n = 292). As an example, for the MVPA and ScT combination, the “do not meet” group (n = 286) included all other adherence patterns, such as meeting MVPA only, ScT only, Sleep only, MVPA and Sleep, ScT and Sleep, or none of the three.

Figs 2 and 3, along with S2 Table, show the differences in physical fitness scores by patterns of 24-h MG adherence. Total physical fitness scores were significantly higher among children who met the MVPA guideline (F [1, 300] = 10.3, P = 0.001, d = 0.47), both the MVPA and ScT guidelines (F [1, 301] = 7.6, P = 0.006, d = 0.63), and both the MVPA and Sleep guidelines (F [1, 301] = 11.6, P < 0.001, d = 0.65), or with all three guidelines (F [1, 302] = 4.8, P = 0.029, d = 0.59), compared to those who did not meet these respective patterns.

Regarding individual physical fitness components in the sit-up test, children who complied with the MVPA guideline or with both the MVPA and ScT guidelines showed significantly higher scores (F [1, 300] = 6.3, P = 0.012, d = 0.37; F [1, 301] = 6.4, P = 0.012, d = 0.58, respectively) than those who did not meet these guidelines. In the repetitive side jump test, children who complied with the MVPA guideline, both the MVPA and ScT guidelines, or both the MVPA and Sleep guidelines showed significantly higher scores (F [1, 300] = 3.9, P = 0.049, d = 0.29; F [1, 301] = 5.1, P = 0.025, d = 0.51; F [1, 301] = 4.4, P = 0.011, d = 0.40, respectively) than those who did not meet these guidelines. In the 20-meter shuttle run test, children who complied with the MVPA guideline, both the MVPA and ScT guidelines, or both the MVPA and Sleep guidelines showed significantly higher scores (F [1, 300] = 15.9, P < 0.001, d = 0.59; F [1, 301] = 4.0, P = 0.047, d = 0.46; F [1, 301] = 8.2, P = 0.004, d = 0.54, respectively) than those who did not meet these guidelines. In the 50-meter sprint, children who complied with the MVPA guideline, both the MVPA and ScT guidelines, both the MVPA and Sleep guidelines, or with all three guidelines showed significantly higher scores (F [1, 300] = 10.3, P = 0.001, d = 0.46; F [1, 301] = 8.4, P = 0.004, d = 0.66; F [1, 301] = 9.5, P = 0.002, d = 0.59; F [1, 302] = 5.5, P = 0.019, d = 0.63, respectively) than those who did not meet these guidelines. In the standing long jump test, children who complied with the MVPA guideline, both the MVPA and ScT guidelines, both the MVPA and Sleep guidelines, or with all three guidelines showed significantly higher scores (F [1, 300] = 5.6, P = 0.019, d = 0.35; F [1, 301] = 10.0, P = 0.002, d = 0.72; F [1, 301] = 10.2, P = 0.002, d = 0.61; F [1, 302] = 6.6, P = 0.011, d = 0.69, respectively) than those who did not meet these guidelines. In the softball throw test, children who complied with the MVPA and Sleep guidelines showed significantly higher scores (F [1, 301] = 4.5, P = 0.036, d = 0.40) than those who did not meet these guidelines.

## Discussion

In the present study, we examined the differences in total fitness and a variety of physical fitness component scores by 24-h MG adherence patterns among Japanese elementary school children, using standardized physical fitness test scores. Total physical fitness scores and many individual fitness components—including trunk muscle strength and endurance, agility, cardiorespiratory fitness, speed, and explosive power—were higher among those who met the MVPA guideline. Importantly, greater differences were observed when MVPA adherence was accompanied by adherence to either the ScT or Sleep guideline. This study adds to existing evidence on the importance of comprehensive adherence to the 24-h MG for promoting physical fitness in children. To our knowledge, this is the first study in Japan to examine differences in physical fitness by 24-h MG adherence patterns using all eight components of the standardized physical fitness test.

Our results are generally consistent with those of previous large-scale studies [27,28] that examined the relationship between total physical fitness and adherence to the 24-h MG. These studies reported that meeting the MVPA guideline, meeting combinations of MVPA with either screen time or sleep guidelines, and meeting all three guidelines were associated with higher levels of overall and individual physical fitness components. One study that examined individuals aged 13–22 years reported significantly higher total fitness scores among those who adhered only to the ScT guideline [28]. In the present study, no significant difference in total fitness scores was observed between children who met and did not meet the ScT guideline. Previous studies have shown that recreational screen time tends to increase with age [32,45], which may partly explain the discrepancy between the two studies due to differences in the age range of participants.

Consistent with the present study, a previous study involving elementary school children also found no association between handgrip strength and adherence to the 24-h MG for muscle strength [30]. Since handgrip strength has been reported to substantially increase after the age of 12 years [46], this lack of association may be explained by the younger age of the participants. Furthermore, a study involving children aged 11–16 years reported a significant association between adherence to the MVPA guideline and handgrip strength [27]. In contrast, trunk muscle strength and endurance, as assessed by the sit-up, were higher among children who met the MVPA guideline, and this association was further strengthened when children adhered to both the MVPA and ScT guidelines. This finding supports previous research suggesting that prolonged ScT is associated with reduced abdominal and trunk muscle strength [47]; musculoskeletal problems in the lumbar region associated with excessive ScT [48,49] may partly account for this relationship. Therefore, in addition to promoting adherence to MVPA, appropriate management of ScT may contribute to improving trunk muscle strength and endurance.

For flexibility, no significant differences were observed between adherence patterns to the 24-h MG, which is consistent with the findings of a previous study conducted among Japanese children [30]. One possible explanation for the absence of a significant relationship is that flexibility may be more strongly influenced by genetic factors than other fitness components. A twin study involving participants aged 6–18 years reported that approximately 80% of the individual differences in flexibility (sit-and-reach) were explained by genetic factors, which suggests that flexibility may reflect anatomically determined traits that are largely regulated by heredity [50].

Agility and speed were assessed using the repetitive side jump and the 50-meter sprint, respectively. Children who met the MVPA guideline demonstrated significantly higher scores in both tests than those who did not. When effect sizes were compared, these associations were further strengthened among those who adhered to both the MVPA and ScT guidelines. Long-term use of digital devices promotes forward head posture in children [51]. In adults, forward head posture has been associated with postural imbalance, decreased postural control, morphological alterations in the thoracic cage, and impaired respiratory function [52,53]. Therefore, prolonged screen time exposure may have been associated with similar postural deterioration, potentially related to lower agility and speed. However, to our knowledge, direct evidence linking screen time-induced posture changes to motor function in children is lacking and warrants further investigation.

The findings of the present study on cardiorespiratory fitness, as assessed by the 20-meter shuttle run, are generally consistent with those of previous studies, which reported positive associations with the MVPA guideline alone [30] or MVPA combined with Sleep guidelines [26,27]. Although previous research has demonstrated associations between prolonged ScT and lower cardiorespiratory fitness [54,55], no significant difference was observed in cardiorespiratory fitness between children who met and those who did not meet the ScT guideline in the present study. This discrepancy may be due to differences in the age of participants. A previous study reported stronger associations between ScT and cardiorespiratory fitness after adolescence [19], whereas our participants were younger children.

Explosive power, as assessed by the standing long jump, was significantly higher among children who met the MVPA guideline, as well as among those who adhered to the MVPA guideline in combination with the ScT and/or Sleep guidelines, compared to those who did not meet the guidelines. Notably, the largest effect size was observed when adherence to the ScT guideline was added to MVPA adherence, highlighting the importance of limiting screen time. Standing long jump performance depends not only on lower limb muscle strength but also on the latissimus dorsi and trapezius muscles, both of which originate from the spinal column [56]. Prolonged ScT has been associated with issues in the neck and back regions, such as musculoskeletal discomfort or pain [57–59]. Thus, extended ScT may suggest that it negatively affects the function of these muscles, ultimately impairing standing long jump performance.

Explosive power and dexterity, assessed by the softball throw, were positively associated with adherence to both the MVPA and Sleep guidelines. The softball throw is among the physical fitness components that have declined most dramatically among Japanese children compared with the 1980s. For example, in boys, the average throw distance at age 7 declined from 15.4 m (1980) to 11.4 m (2021), and at age 11 from 34.0 m (1980) to 25.4 m (2021). In girls, the average throw distance at age 7 declined from 8.8 m (1980) to 7.4 m (2021), and at age 11 from 20.5 m (1980) to 15.2 m (2021) [46]. One possible explanation for this trend is the declining number of children participating in baseball [60], a sport that involves overhand throwing skills similar to those required for the softball throw. Although both the standing long jump and softball throw assess explosive power, the latter also requires dexterity and coordination. Therefore, adherence to the MVPA guideline, which includes physical activity and sports participation, might improve softball throw performance by enhancing overall motor coordination and dexterity during childhood [61,62]. In addition, adherence to the Sleep guideline positively influences the acquisition and consolidation of gross motor skills [63,64], which may further support better performance.

This study contributes to the growing body of literature by examining how adherence to the 24-h MG relates to various aspects of physical fitness using a standardized eight-item test among Japanese children. The results underscore the potential benefits of combining MVPA with either ScT or Sleep adherence in promoting total and multiple aspects of physical fitness. These findings further suggest that, in addition to promoting MVPA among children, strategies that encourage appropriate ScT and Sleep behaviors based on the 24-h MG—both in school settings and at home—may be effective in enhancing children’s physical fitness.

The present study has several limitations that should be considered. First, data were collected from a single elementary school with a relatively small sample size. Additionally, the total physical fitness scores were approximately 5 points lower than the national average at each grade level, limiting the generalizability of the results. This difference may be partially attributable to the characteristics of the participating school and region. In Yokohama City, where the study was conducted, children’s total physical fitness scores tend to be lower than the national average [65]. Furthermore, the participating school is a new type of “compulsory education school,” which may have slightly different physical fitness trends compared with general schools. Future studies should include multiple schools and larger sample sizes to enhance the validity of these findings. Second, MVPA, ScT, and Sleep data were obtained via self- or parent-assisted questionnaire, which may be subject to recall bias. In addition, children in grades 4–6 completed the questionnaires by themselves, whereas those in grades 1–3 did so with parental assistance. Differences in the response method may have influenced the results. Future studies using objective measures of physical activity and sleep are warranted to further validate these findings. Third, only standardized scores converted to a 10-point scale were obtained for each physical fitness test item, and raw measurement values were not collected. Using Japan-specific standardized scores makes international comparisons difficult; therefore, future studies should collect raw measurement values as well. In addition, although we received an official email response from the Japan Sports Agency, which oversees the physical fitness test, confirming that the evaluation criteria were developed using percentile values based on data from Japanese children, no publicly available documents describing the detailed process of this development were found. This should be considered a limitation when interpreting the findings. Fourth, owing to missing response data, only 65% of the total dataset was included in the final analysis, which may have influenced the results. Finally, as this was a cross-sectional study, causal relationships between adherence to the 24-h MG and physical fitness cannot be inferred. Although this study provides initial evidence of the relationship between adherence to the 24-h MG and individual fitness components, it does not fully explain the underlying reasons certain fitness components improved or remained stable while others declined. Future research employing detailed longitudinal designs is necessary to explore these questions.

## Conclusion

Adherence to the MVPA guideline was strongly associated with higher total physical fitness and most individual physical fitness components among elementary school-aged children. Furthermore, adherence to the MVPA guideline in combination with ScT and/or Sleep guidelines further strengthened these associations with specific components of physical fitness.

## Supporting information

S1 Table Standardized fitness test score table by item for Japanese elementary schools. (PDF)

S2 Table Differences in physical fitness scores by 24-hour movement guidelines adherence patterns. (PDF)
