# Supplementary material for: Differences in physical fitness levels by adherence to the 24-hour movement guidelines among Japanese elementary school children
Source: PLoS One. 2025 Dec 3;20(12):e0337972. doi: 10.1371/journal.pone.0337972 (PMC12674516; doi:10.1371/journal.pone.0337972)
Supplement: S1 Table — (PDF) [file pone.0337972.s001.pdf]

S1 Table. Standardized fitness test score table by item for Japanese elementary schools.

<Boy>

Score	Handgrip strength (kg)	Sit-up (times)	Sit-and-reach (cm)	Repetitive side jump (points)	20-meter shuttle run (times)	50-meter sprint (sec)	Standing long jump (cm)	Softball throw (m)
10	≥ 26	≥ 26	≥ 49	≥ 50	≥ 80	≤ 8.0	≥ 192	≥ 40
9	23-25	23-25	43-48	46-49	69-79	8.1-8.4	180-191	35-39
8	20-22	20-22	38-42	42-45	57-68	8.5-8.8	168-179	30-34
7	17-19	18-19	34-37	38-41	45-56	8.9-9.3	156-167	24-29
6	14-16	15-17	30-33	34-37	33-44	9.4-9.9	143-155	18-23
5	11-13	12-14	27-29	30-33	23-32	10.0-10.6	130-142	13-17
4	9-10	9-11	23-26	26-29	15-22	10.7-11.4	117-129	10-12
3	7-8	6-8	19-22	22-25	10-14	11.5-12.2	105-116	7-9
2	5-6	3-5	15-18	18-21	8-9	12.3-13.0	93-104	5-6
1	≤ 4	≤ 2	≤ 14	≤ 17	≤ 7	≥ 13.1	≤ 92	≤ 4

<Girl>

Score	Handgrip strength (kg)	Sit-up (times)	Sit-and-reach (cm)	Repetitive side jump (points)	20-meter shuttle run (times)	50-meter sprint (sec)	Standing long jump (cm)	Softball throw (m)
10	≥ 25	≥ 23	≥ 52	≥ 47	≥ 64	≤ 8.3	≥ 181	≥ 25
9	22-24	20-22	46-51	43-46	54-63	8.4-8.7	170-180	21-24
8	19-21	18-19	41-45	40-42	44-53	8.8-9.1	160-169	17-20
7	16-18	16-17	37-40	36-39	35-43	9.2-9.6	147-159	14-16
6	13-15	14-15	33-36	32-35	26-34	9.7-10.2	134-146	11-13
5	11-12	12-13	29-32	28-31	19-25	10.3-10.9	121-133	8-10
4	9-10	9-11	25-28	25-27	14-18	11.0-11.6	109-120	6-7
3	7-8	6-8	21-24	21-24	10-13	11.7-12.4	98-108	5
2	4-6	3-5	18-20	17-20	8-9	12.5-13.2	85-97	4
1	≤ 3	≤ 2	≤ 17	≤ 16	≤ 7	≥ 13.3	≤ 84	≤ 3

Source: Ministry of Education, Culture, Sports, Science and Technology (MEXT). New Physical Fitness Test Implementation Guidelines. 1999. Available from: https://www.mext.go.jp/sports/b_menu/sports/mcatetop03/list/detail/1408001.htm (In Japanese)
