# Supplementary material for: Differences in physical fitness levels by adherence to the 24-hour movement guidelines among Japanese elementary school children
Source: PLoS One. 2025 Dec 3;20(12):e0337972. doi: 10.1371/journal.pone.0337972 (PMC12674516; doi:10.1371/journal.pone.0337972)
Supplement: S2 Table — (PDF) [file pone.0337972.s002.pdf]

S2 Table. Differences in physical fitness scores by 24-hour movement guidelines adherence patterns.

		Total fitness score				Handgrip strength				Sit-up				Sit-and-reach				Repetitive side jump				20-meter shuttle run				50-meter sprint				Standing long jump				Softball throw			
	N	Mean	95% CI	d	*P*	Mean	95% CI	d	*P*	Mean	95% CI	d	*P*	Mean	95% CI	d	*P*	Mean	95% CI	d	*P*	Mean	95% CI	d	*P*	Mean	95% CI	d	*P*	Mean	95% CI	d	*P*	Mean	95% CI	d	*P*
**MVPA**																																					
Meet	59	47.2	45.4-49.1	0.47	**0.001***	5.4	5.1-5.7	0.04	0.787	7.0	6.5-7.4	0.37	**0.012***	6.0	5.5-6.5	0.05	0.738	5.4	4.9-5.8	0.29	**0.049***	6.1	5.8-6.5	0.59	**<.001***	6.3	5.9-6.6	0.46	**0.001***	5.9	5.5-6.2	0.35	**0.019***	5.3	4.9-5.6	0.28	0.056
Do not meet	248	43.9	43.0-44.7			5.4	5.2-5.5			6.4	6.2-6.6			6.1	5.8-6.3			4.9	4.6-5.1			5.3	5.1-5.5			5.6	5.5-5.8			5.3	5.2-5.5			4.9	4.7-5.1		
**ScT**																																					
Meet	94	45.2	43.7-46.7	0.14	0.283	5.3	5.1-5.6	0.07	0.607	6.6	6.3-7.0	0.15	0.267	6.1	5.7-6.5	0.03	0.805	5.1	4.7-5.4	0.10	0.441	5.7	5.4-6.0	0.19	0.139	5.8	5.6-6.1	0.10	0.433	5.6	5.3-5.9	0.13	0.337	5.0	4.7-5.3	0.02	0.867
Do not meet	213	44.2	43.2-45.2			5.4	5.3-5.6			6.4	6.2-6.6			6.0	5.8-6.3			4.9	4.7-5.1			5.4	5.2-5.6			5.7	5.5-5.9			5.4	5.2-5.6			5.0	4.8-5.1		
**Sleep**																																					
Meet	184	44.2	43.1-45.3	0.11	0.374	5.4	5.2-5.6	0.02	0.888	6.3	6.1-6.6	0.23	0.064	6.2	5.9-6.5	0.20	0.106	4.8	4.5-5.1	0.21	0.090	5.4	5.2-5.6	0.19	0.124	5.7	5.5-6.0	0.01	0.958	5.4	5.2-5.6	0.02	0.855	4.9	4.7-5.1	0.09	0.471
Do not meet	123	45.0	43.7-46.3			5.4	5.2-5.6			6.7	6.4-7.0			5.8	5.4-6.2			5.2	4.9-5.5			5.6	5.4-5.9			5.8	5.5-6.0			5.5	5.2-5.7			5.0	4.8-5.3		
**MVPA and ScT**																																					
Meet	21	48.7	45.6-51.8	0.63	**0.006***	5.2	4.7-5.7	0.14	0.528	7.3	6.6-8.0	0.58	**0.012***	5.9	5.1-6.8	0.06	0.806	5.8	5.0-6.5	0.51	**0.025***	6.1	5.5-6.7	0.46	**0.047***	6.6	6.0-7.2	0.66	**0.004***	6.4	5.8-7.0	0.72	**0.002***	5.4	4.8-5.9	0.31	0.179
Do not meet	286	44.2	43.4-45.0			5.4	5.3-5.5			6.4	6.2-6.6			6.0	5.8-6.3			4.9	4.7-5.1			5.4	5.3-5.6			5.7	5.5-5.8			5.4	5.2-5.5			5.0	4.8-5.1		
**MVPA and Sleep**																																					
Meet	32	48.6	46.1-51.1	0.65	**<.001***	5.7	5.3-6.1	0.33	0.085	7.0	6.4-7.5	0.35	0.064	6.0	5.4-6.7	0.00	0.988	5.6	5.0-6.2	0.40	**0.011***	6.2	5.7-6.7	0.54	**0.004***	6.5	6.0-6.9	0.59	**0.002***	6.2	5.7-6.7	0.61	**0.002***	5.4	5.0-5.9	0.40	**0.036***
Do not meet	275	44.0	43.2-44.9			5.3	5.2-5.5			6.4	6.2-6.6			6.0	5.8-6.3			4.9	4.7-5.1			5.4	5.2-5.6			5.7	5.5-5.8			5.3	5.2-5.5			4.9	4.8-5.1		
**ScT and Sleep**																																					
Meet	69	44.2	42.3-46.0	0.06	0.677	5.3	5.0-5.6	0.14	0.340	6.4	6.0-6.8	0.08	0.574	6.2	5.7-6.7	0.11	0.444	5.0	4.5-5.4	0.02	0.890	5.5	5.1-5.9	0.02	0.867	5.7	5.3-6.0	0.10	0.515	5.4	5.0-5.7	0.06	0.710	4.8	4.5-5.1	0.17	0.246
Do not meet	238	44.6	43.7-45.5			5.4	5.3-5.6			6.5	6.3-6.7			6.0	5.7-6.2			4.9	4.7-5.2			5.5	5.3-5.7			5.8	5.6-6.0			5.5	5.3-5.6			5.0	4.9-5.2		
**All three**																																					
Meet	15	48.5	44.8-52.2	0.59	**0.029***	5.3	4.7-5.9	0.11	0.679	7.0	6.2-7.9	0.37	0.170	6.1	5.1-7.1	0.04	0.889	5.8	4.9-6.7	0.51	0.057	6.2	5.5-6.9	0.52	0.054	6.6	5.9-7.3	0.63	**0.019***	6.4	5.6-7.2	0.69	**0.011***	5.2	4.5-5.8	0.15	0.573
Do not meet	292	44.3	43.5-45.1			5.4	5.3-5.5			6.5	6.3-6.6			6.0	5.8-6.3			4.9	4.7-5.1			5.4	5.3-5.6			5.7	5.6-5.9			5.4	5.2-5.6			5.0	4.8-5.1		

MVPA: moderate-to-vigorous physical activity: ScT: screen time: Sleep: sleep duration. Total fitness score ranges from 8-80, with a score range of 1-10 for each fitness component.

Comparisons were conducted between children who met a specific recommendation pattern and those who did not. For the labels "MVPA," "ScT," and "Sleep," each category includes all children meeting the respective guideline, regardless of whether they also met the other guidelines; adherence to the other guidelines was included as covariates in the model to minimize potential confounding. Asterisks indicate statistical significance: $P < 0.05$ (*). As an example, for the MVPA and ScT combination, the "do not meet" group (n = 286) included all other adherence patterns, such as meeting MVPA only, ScT only, Sleep only, MVPA and Sleep, ScT and Sleep, or none of the three.
